# Perforation by Proctoscope

**Published:** 1913-02

**Authors:** 


PERFORATION BY PROCTOSCOPE.—O. Retzloff, *Münch. Med. Woch.,* July 23, 1912, reports a case which recovered after suture.

